# Epidemiology and management of gout in Taiwan: a nationwide population study

**DOI:** 10.1186/s13075-015-0522-8

**Published:** 2015-01-23

**Authors:** Chang-Fu Kuo, Matthew J Grainge, Lai-Chu See, Kuang-Hui Yu, Shue-Fen Luo, Weiya Zhang, Michael Doherty

**Affiliations:** Division of Rheumatology, Orthopaedics and Dermatology, School of Medicine, University of Nottingham, Academic Rheumatology, Clinical Sciences Building, Nottingham City Hospital, Hucknall Road, Nottingham, NG5 1PB UK; Division of Rheumatology, Allergy and Immunology, Chang Gung Memorial Hospital, 5, Fuxing street, Taoyuan, 333 Taiwan; Division of Epidemiology and Public Health, School of Medicine, University of Nottingham, Clinical Sciences Building, Nottingham City Hospital, Hucknall Road, Nottingham, NG5 1PB UK; Department of Public Health, College of Medicine, Chang Gung University, 259, Wenhua 1st road, Taoyuan, 333 Taiwan; Biostatistics Core Laboratory, Molecular Medicine Research Centre, Chang Gung University, 259, Wenhua 1st road, Taoyuan, 333 Taiwan

## Abstract

**Introduction:**

Gout is the most common inflammatory arthritis worldwide and is the only type of chronic arthritis that potentially can be ‘cured’. However, data on gout incidence, prevalence and management, assessed at multiple time points in the same population, are sparse, particularly in Asian populations. The aim of this study was to describe trends in the epidemiology of gout in the general population of Taiwan.

**Methods:**

The National Health Insurance Research Database was used to identify patients with gout and to estimate the prevalence and incidence of gout for each calendar year from 2005 to 2010. The pattern of gout management was also examined.

**Results:**

Of 23,371,362 beneficiaries in 2010, there were 1,458,569 prevalent and 56,595 incident cases of gout, giving a prevalence of 6.24% (95% confidence interval (CI), 6.23% to 6.25%) and an incidence of 2.74 (95% CI, 2.72 to 2.76) per 1,000 person-years. The annual percentage change (APC) of the standardised prevalence was −0.7% (95% CI, −1.7% to 0.3%; *P* = 0.14), suggesting that the prevalence of gout was essentially the same throughout the study period. However, The APC of incidence was −13.4 (95% CI, −16.1 to −10.6) between 2005 and 2007 and −2.1 (95% CI, −10.4 to 7.1) between 2007 and 2010. Regions with the highest prevalence and incidence were eastern coastal counties and offshore islets, where indigenous people are clustered. Among prevalent gout cases in 2010, only 22.93% (95% CI, 22.87% to 23.00%) were prescribed urate-lowering treatment (ULT), which remained unchanged between 2005 and 2010 at an APC of 0.0 (95% CI, −3.8 to 4.0). Uricosuric agents were more commonly prescribed than xanthine oxidase inhibitors in Taiwan.

**Conclusions:**

In Taiwan, 1 in 16 people have gout. Whereas the incidence has decreased recently, the prevalence remains unchanged. Management of gout in Taiwan is poor, with only one in five affected people being treated with ULT.

**Electronic supplementary material:**

The online version of this article (doi:10.1186/s13075-015-0522-8) contains supplementary material, which is available to authorized users.

## Introduction

Gout is the most common inflammatory arthritis and constitutes a rising disease burden worldwide [1]. Gout is defined by the presence of monosodium urate crystals which deposit as a consequence of chronic elevation of serum urate above the saturation point for urate crystal formation. The main accepted clinical consequences of gout are recurrent episodes of acute painful arthritis (gout ‘attacks’), irreversible peripheral joint damage, subcutaneous tophi, urate nephropathy and urolithiasis [2]. Reducing and maintaining serum urate levels below the saturation point can prevent further crystal formation and promote dissolution of existing urate crystals, thereby eliminating the pathogenic agents and leading to ‘cure’. However, despite its high prevalence and relatively well-understood pathogenesis, as well as the existence of definitive urate-lowering therapy (ULT), gout is largely ignored by physicians in primary care [3], who very often treated it as an acute illness rather than as a chronic disease with major adverse consequences [4-6].

The prevalence and incidence of gout have a distinct geographical and racial distribution [7]. In a recent gout study in the United Kingdom, researchers found a prevalence of 2.49% and an incidence of 1.77 per 1,000 person-years in 2012, with both figures being higher than earlier estimates [8]. In the United States, the National Health and Nutrition Examination Survey team also found a high prevalence of gout, which was 3.9% between 2007 and 2008 [9]. Contemporary data on gout epidemiology are relatively scant in other countries. Taiwan is one of the countries with the highest prevalence of gout worldwide [10-17]. In a recent nationwide survey (the Nutrition and Health Survey in Taiwan), researchers reported gout prevalence data of 8.2% in men and 2.3% in women in the period from 2005 to 2008 [17], which is close to the estimates for 2004 based on information derived from the National Health Insurance Research Database (NHIRD) [18]. In particular, Taiwanese aborigines have a very high prevalence of gout [11,12], which is shared with their genetically related Polynesian cousins [19], the Māori and indigenous Oceania/Pacific Islander populations [20]. The incidence of gout has not been estimated in Taiwan previously, and the current standard of care in Taiwan as reflected by the use of ULT has not been examined to date.

Therefore, we undertook this study to examine the prevalence and incidence of gout and the patterns of gout management between 2005 and 2010 using the NHIRD, which contains comprehensive information on diagnoses, prescriptions and hospitalisations of essentially the entire population in Taiwan.

## Methods

The study was approved by the institutional review board of Chang Gung Memorial Hospital (approval number 101-2178C). All data in this study were anonymised; therefore, the need for patient consent was waived.

### Source of data and study population

In this study, we used data from the NHIRD, which contains registration files and original claims data from approximately 28.75 million (living and deceased) beneficiaries registered in the database between March 1995 and the end of 2010. We requested from the National Health Research Institute (the data holder of NHIRD) a tailor-made dataset containing data of all patients with a diagnosis of gout. Denominator data were based on the Registry of Beneficiaries, a part of NHIRD with records of the demographics, insurance status, residence and socioeconomic data of all beneficiaries. However, ethnicity data were not available. Our study comprised all beneficiaries registered continuously between 1 January 2005 and 31 December 2010.

### Case definition of gout

The primary case definition of gout was having a physician-recorded primary diagnosis of gout (International Classification of Diseases, Ninth Revision, code 274.x) at either an outpatient or emergency visit. By searching the entire NHIRD, we obtained data on all patients with gout recorded between 1995 and 2010.

### Estimation of prevalence and incidence

Prevalent cases of gout were defined as individuals who had at least one record of a primary diagnosis of gout within the 10-year period before 1 July of each calendar year. The denominator for prevalence estimation (the eligible population) for each calendar year included all individuals registered on 1 July of each calendar year. Prevalence was calculated using the number of prevalent cases of gout divided by the eligible population in the specified calendar year.

Incident cases of gout defined as those patients who had no evidence of gout or use of ULT within the 10-year period prior to 1 January of each calendar year but who developed gout during that year. We chose to fix the observational period to 10 years for two reasons: (1) The NHIRD does not have data going back further than 10 years for beneficiaries registered in 2005, and (2) the majority of prevalent gout cases can be identified within a 10-year observation period, according to a previous cohort study [21]. To be eligible to be considered as incident gout cases, beneficiaries had to have at least a 1-year registration period prior to 1 January of each calendar year. For the incidence of gout, we constructed at-risk cohorts for each calendar year, comprising all individuals registered during the given calendar year who had no history of a gout diagnosis before 1 January of that year. Incidence was calculated using the number of incident gout cases during a calendar year as the numerator and the total person-years in an at-risk population accumulated during that same year as the denominator.

Prevalence and incidence were calculated for 21 cities and/or counties in Taiwan (termed *regions* hereinafter): Taipei city, Taipei county, Keelung city, Taoyuan county, Hsinchu city and county, Yilan county, Miaoli county, Taichung city, Taichung county, Changhua county, Yunlin county, Nantou county, Chiayi city and county, Tainan city, Tainan county, Kaohsiung city, Kaohsiung county, Pingtung county, Hualien county, Taitung county and offshore islets (Penghu, Kinmen and Lienchiang counties). To remove the effect of different age and sex structures in these regions, we standardised prevalence and incidence with respect to the overall population structure of 2010. We used choropleth maps to represent geographic variations in gout incidence and prevalence between different regions of Taiwan.

### Pattern of medication use

For each calendar year from 2005 to 2010, we ascertained the proportion of prevalent gout cases in which the patient received ULT (allopurinol, benzbromarone, probenecid or sulfinpyrazone).

### Trends of prevalence, incidence and management of gout

To determine the trends in prevalence, incidence and management of gout, we calculated age- and sex-standardised prevalence, incidence of gout and pattern of ULT prescribing in each calendar year from 2005 to 2010 with the population structure in 2010 used as the reference.

### Statistical analysis

The 95% confidence intervals (CIs) for prevalence and incidence were derived on the basis of the assumption of a Poisson distribution for the observed number of prevalent and incident cases. We used the Joinpoint Regression Program (version 4.0.4; National Cancer Institute, Bethesda, MD, USA) to estimate trends in the prevalence and incidence of gout. We used the Bayesian information criterion in the program to generate different numbers of ‘join points’ in time when the linear trend of prevalence and incidence of gout changed significantly and to determine the best-fit data series [22]. A maximum of two join points was used to determine statistical significance for trend. Annual percentage changes (APCs) for each segment were calculated. The significance level was set at 0.05. All statistical analyses were performed using SAS statistical software, version 9.3 (SAS Institute, Cary, NC, USA).

## Results

### Prevalence and incidence of gout in 2010

**Figure 1:**
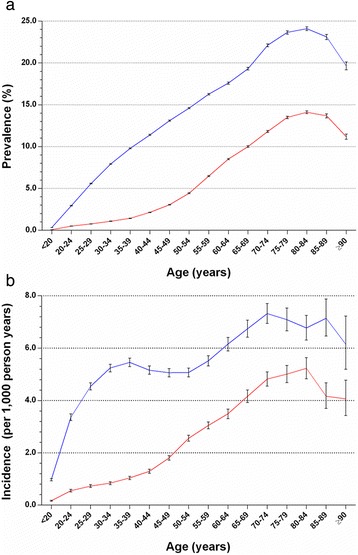
**Age-specific prevalence (a) and incidence (b) of gout in Taiwan in 2010.** Blue: men; red: women.

Of 23,371,362 beneficiaries (men: 49.56%) included within the NHIRD in 2010, 1,458,569 prevalent cases of gout were identified, giving a prevalence of 6.24% (95% CI, 6.23% to 6.25%). Men had a significantly higher prevalence of gout (9.34%; 95% CI, 9.32% to 9.36%) than women (3.20%; 95% CI, 3.19% to 3.21%). Overall, the prevalence of gout was 2.9-fold higher in men than in women. This sex difference was observed in all age groups, with a male-to-female ratio of 4.5 in individuals younger than 20 years, peaking at 7.3 in those ages 30 to 34 years and then decreasing thereafter. Gout was rare in people younger than 20 years of age, and it increased with age, reaching a peak in the 80- to 84-year-old age band (Figure 1a).

There were a total 20,677,590 person-years of follow-up in 2010, during which 56,595 incident cases of gout were identified (overall incidence, 2.74 (95% CI, 2.72 to 2.76) per 1,000 person-years). Men had a higher incidence of gout (4.10 (95% CI, 4.06 to 4.14) per 1,000 person-years) than women (1.49 (95% CI, 1.47 to 1.52) per 1,000 person-years). As shown in Figure 1b, the incidence of gout in men was low in those younger than 20 years of age but increased rapidly until ages 35 to 39 years. After a relative plateau in gout incidence during middle age, gout incidence again showed a sharp increase, reaching a peak between ages 70 and 74 years. In women, the incidence of gout remained low before the age of 40, but thereafter it started to increase rapidly, reaching a peak incidence at the age band of 80 to 84 years. The greatest male-to-female incidence ratio (6.2) was observed in those ages 30 to 34 years, and thereafter the difference between men and women diminished.

### Prevalence and incidence of gout between 2005 and 2010

**Figure 2:**
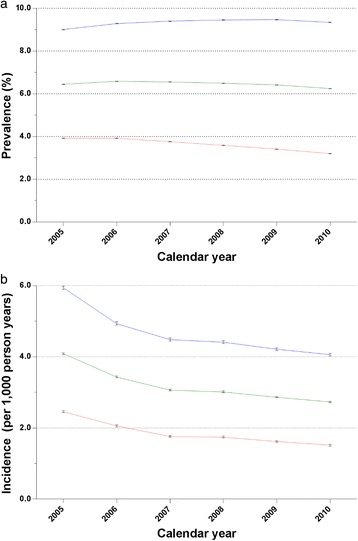
**Differences by sex in the trends of standardised prevalence (a) and incidence (b) of gout in Taiwan between 2005 and 2010.** Blue: men; red: women; green: overall).

Table 1 shows the temporal trends in prevalence and incidence of gout from 2005 to 2010. In general, the standardised estimates were slightly higher than the crude ones, accounting for the fact that the population was aging over time (refer to Additional file 1: Figure S1 for population pyramids of Taiwan in 2005 and Additional file 2: Figure S2 for 2010). The crude prevalence of gout increased 5.6%, but the standardised prevalence fluctuated and decreased by 3.1% over the study period. The annual percentage change of the standardised prevalence was −0.7 (95% CI, −1.7 to 0.3), but this was not statistically significant (*P* = 0.14), suggesting that the prevalence of gout was essentially the same throughout the study period after adjusting for age. There was no significant join point for the trend in gout prevalence. As Figure 2a shows, the temporal trend in prevalence among men and women was not parallel (*P* < 0.001). The annual percentage change was 1.0 (95% CI, 0.1 to 1.9) in men and −4.0 (95% CI, −5.4 to −2.7) in women. The male-to-female ratio increased slightly from 2.3 in 2005 to 2.9 in 2010.

**Table 1:** **Crude and standardised prevalence and incidence of gout from 2005 to 2010**

**Year**	**Prevalence (%)**			**Incidence (per 1,000 person-years)**		
	***N***	**Crude**	**Standardised**	**Person-years**	**Crude**	**Standardised**
**Overall**						
2005	23,000,521	5.91 (5.90 to 5.92)	6.44 (6.43 to 6.45)	20,543,857	3.81 (3.78 to 3.84)	3.93 (3.90 to 3.96)
2006	23,127,946	6.14 (6.13 to 6.15)	6.58 (6.57 to 6.59)	20,559,914	3.21 (3.19 to 3.24)	3.29 (3.27 to 3.31)
2007	23,221,905	6.22 (6.21 to 6.23)	6.55 (6.54 to 6.56)	20,602,277	2.89 (2.86 to 2.91)	2.93 (2.91 to 2.96)
2008	23,315,001	6.27 (6.26 to 6.28)	6.49 (6.48 to 6.50)	20,645,964	2.89 (2.87 to 2.92)	2.91 (2.88 to 2.93)
2009	23,344,259	6.30 (6.29 to 6.31)	6.41 (6.40 to 6.42)	20,666,404	2.80 (2.78 to 2.82)	2.79 (2.76 to 2.81)
2010	23,371,362	6.24 (6.23 to 6.25)	6.24 (6.23 to 6.25)	20,677,590	2.74 (2.72 to 2.76)	2.74 (2.72 to 2.76)
**Men**						
2005	11,550,180	8.35 (8.33 to 8.36)	9.01 (8.99 to 9.02)	10,048,241	5.55 (5.47 to 5.56)	5.64 (5.59 to 5.68)
2006	11,579,907	8.74 (8.72 to 8.76)	9.28 (9.27 to 9.30)	10,001,584	4.68 (4.64 to 4.72)	4.76 (4.71 to 4.80)
2007	11,598,597	8.98 (8.96 to 8.99)	9.40 (9.38 to 9.41)	9,969,808	4.26 (4.22 to 4.30)	4.30 (4.26 to 4.34)
2008	11,615,445	9.16 (9.14 to 9.18)	9.45 (9.43 to 9.47)	9,944,293	4.26 (4.22 to 4.30)	4.26 (4.22 to 4.30)
2009	11,597,439	9.32 (9.30 to 9.34)	9.47 (9.45 to 9.48)	9,907,190	4.16 (4.12 to 4.20)	4.13 (4.09 to 4.17)
2010	11,583,208	9.34 (9.32 to 9.36)	9.34 (9.32 to 9.36)	9,868,937	4.10 (4.06 to 4.14)	4.10 (4.06 to 4.14)
**Women**						
2005	11,450,341	3.46 (3.45 to 3.47)	3.92 (3.91 to 3.93)	10,495,616	2.18 (2.15 to 2.20)	2.37 (2.34 to 2.40)
2006	11,548,039	3.54 (3.53 to 3.55)	3.92 (3.91 to 3.93)	10,558,330	1.82 (1.79 to 1.84)	1.95 (1.92 to 1.97)
2007	11,623,308	3.48 (3.47 to 3.49)	3.76 (3.75 to 3.77)	10,632,469	1.60 (1.57 to 1.62)	1.68 (1.66 to 1.71)
2008	11,699,556	3.40 (3.39 to 3.41)	3.59 (3.57 to 3.60)	10,701,671	1.62 (1.60 to 1.65)	1.67 (1.65 to 1.70)
2009	11,746,820	3.31 (3.30 to 3.32)	3.41 (3.40 to 3.42)	10,759,214	1.55 (1.52 to 1.57)	1.56 (1.54 to 1.58)
2010	11,788,157	3.20 (3.19 to 3.21)	3.20 (3.19 to 3.21)	10,808,653	1.49 (1.47 to 1.52)	1.49 (1.47 to 1.52

In contrast, both the crude and standardised incidences of gout declined over time during this period, with the standardised incidence reducing by 30.3% (Figure 2b). On average, the annual percentage change of gout incidence was −6.7 (95% CI, −10.7 to −2.6), and there was one join point (in 2007). The annual percentage changes of gout incidence were −13.4 (95% CI, −16.1 to −10.6) during the period between 2005 and 2007 and −2.1 (95% CI, −10.4 to 7.1) during the period from 2007 to 2010. Figure 2b shows the trend in gout incidence for men and women (*P* = 0.02), with an annual percentage change of −8.5 (95% CI, −12.6 to −4.3) in men and −5.9 (95% CI, −9.9 to −1.8) in women. However, in both men and women, the trends of gout incidence in the period from 2007 to 2010 were essentially flat, with annual percentage changes (95% CIs) of −3.8 (−12.9 to 6.3) for men and −1.3 (−9.6 to 7.7) for women. The male-to-female ratio of incidence slightly increased from 2.4 in 2005 to 2.7 in 2010.

### Geographic variation in 2010

**Figure 3:**
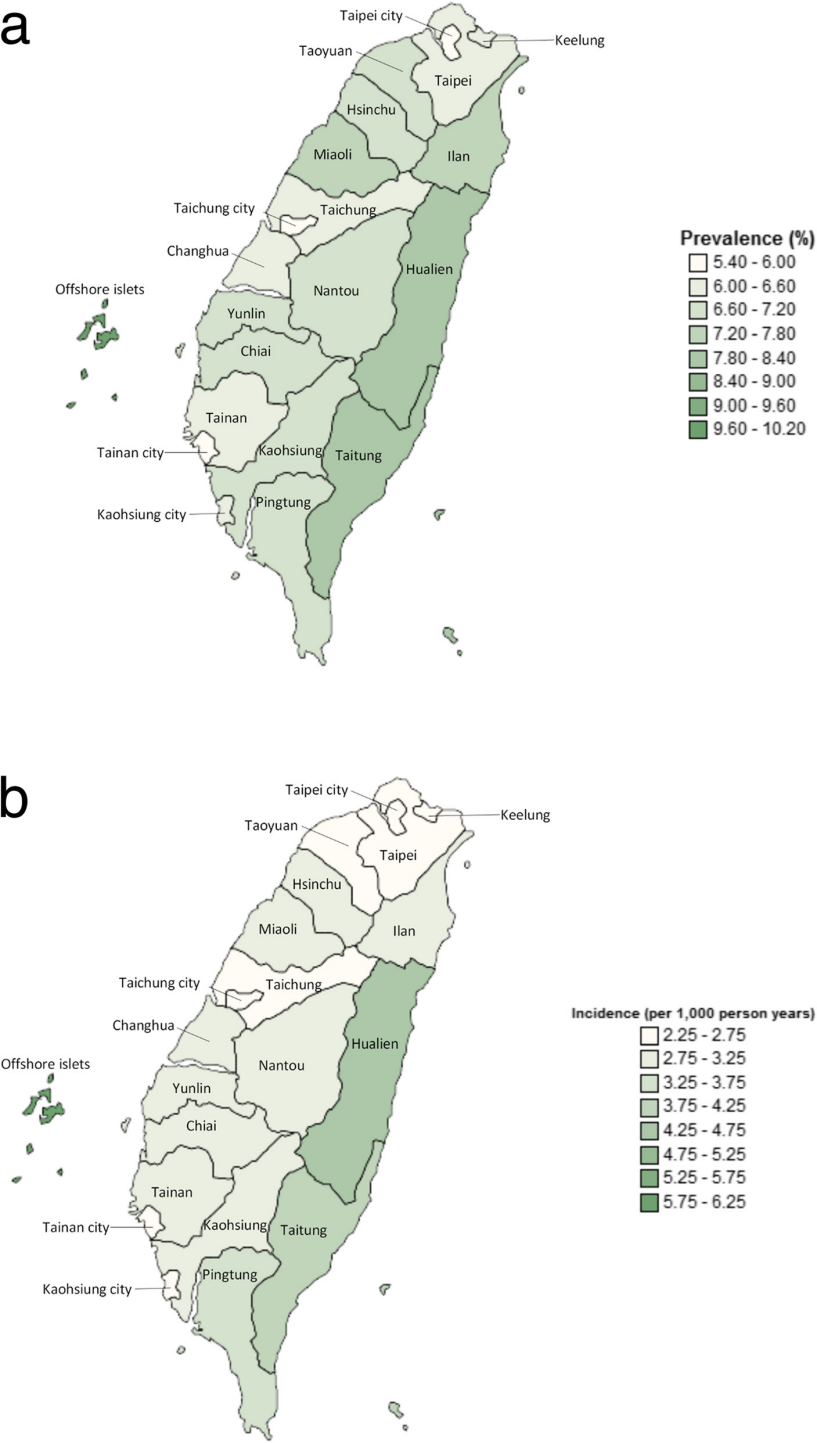
**Geographic variation in the prevalence (a) and incidence (b) of gout in Taiwan in 2010.**

Neither the prevalence nor the incidence of gout was uniform throughout Taiwan. As shown in Figure 3, the standardised prevalence (95% CI) of gout was highest in the eastern coastal counties and offshore islets. The regions with the lowest prevalence of gout were mostly in the urban areas (Taipei city, Taichung city, Tainan city and Kaohsiung city). The regional pattern of gout incidence resembled that of gout prevalence, with a higher incidence in the eastern coastal counties and offshore islets and a lower incidence in the urban areas.

### Management of gout between 2005 and 2010

**Figure 4:**
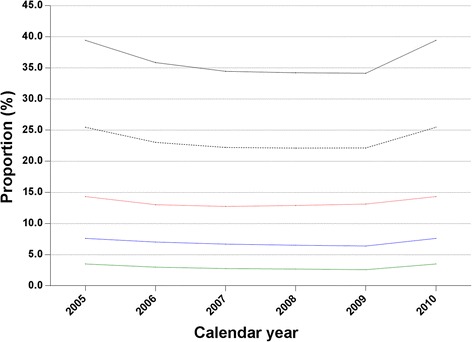
**Secular trends of treatment of gout in Taiwan.** Proportion of prevalent gout cases in which patients received consultations for gout or underwent urate-lowering treatment (black line), only received urate-lowering treatment (black dotted line), received uricosuric agents (red line), received a xanthine oxidase inhibitor (blue line) or received both (green line).

Of the 1,458,569 prevalent gout cases in 2010, approximately one-third of patients had consulted for gout or had been treated with ULT (*n* = 515,004; 35.31% (95% CI, 35.23% to 35.39%)). However, only one in five prevalent gout cases in 2010 involved patients who were treated with ULT (*n* = 334,518; 22.93% (95% CI, 22.87% to 23.00%)). Among patients who received ULT treatment, 60.08% (95% CI, 59.91% to 60.25%) received uricosuric agents alone, 28.54% (95% CI, 28.39% to 28.69%) received a xanthine oxidase inhibitor and 11.38% (95% CI, 11.27% to 11.49%) received both. As shown in Figure 4a, the proportion of prevalent cases in which patients received consultations for gout or were treated by ULT remained low during the whole study period, with an APC of −0.9 (95% CI, −6.2 to 4.7). Similarly, the proportion of patients who received ULT did not change during this time, with an APC of 0.0 (−3.8 to 4.0).

## Discussion

In this nationwide population study covering the entire 23 million population of Taiwan, about 1 in 16 residents of Taiwan was affected by gout during the study period. Whereas the incidence decreased, the prevalence of gout was fairly stable over the period between 2005 and 2010. Despite the small size of Taiwan (only 36,193 km^2^), both the prevalence and incidence of gout showed marked geographical variation. This regional variation in gout generally coincided with the distribution of aboriginal people in Taiwan, who primarily reside in eastern counties and rural areas. Although gout is one of the most common chronic diseases in Taiwan, it seems that the management of gout remains poor, with only one-third of people with gout receiving medical attention and only one-fifth being given ULT. Unfortunately, this suboptimal care did not change over the study period, despite the publication of national and international guidelines on gout management during this time.

The trends of gout prevalence in Taiwan have not been robustly examined until now. In general, surveys conducted in the 1990s [10-14] reported a lower prevalence of gout than those conducted in the 2000s [15-18]. The National Nutrition and Health Survey in Taiwan undertaken in the period between 1993 and 1996 found the prevalence of gout to be 4.74% in men and 2.19% in women [14], whereas the same survey conducted between 2005 and 2008 found the prevalence of gout to be 8.21% in men and 2.33% in women [17]. However, these estimates were based on patients’ self-reported gout and were not standardised for age structure, despite use of stratified probability sampling considering age, sex, geographical region and ethnicity in the respective study periods. In our present study, we found that the crude prevalence of gout increased over time but that the age- and sex-standardised prevalence remained stable between 2005 and 2010. There were different trends in gout prevalence in men and women, with the prevalence in men reaching a plateau after 2007, whereas the prevalence in women continued to decrease throughout the study period.

Several caveats should be considered before drawing conclusion about trends in gout prevalence in Taiwan. First, most previous studies were based on patients’ self-reported gout [10-17], which has inherent bias. For example, in the Atherosclerosis Risk in Communities (ARIC) cohorts [23], only 73% of patients with gout diagnosed by this means in 2000 also reported gout in the follow-up questionnaire in 2003, and only 65% reported gout in the 2003 and 2007 questionnaires. In a Dutch study, researchers found that only 64% of patients with gout self-reported it after just 6 months of follow-up [24]. Furthermore, an earlier study found that only 44% of self-reported gout cases had this confirmed by medical chart review and physician interview [25]. These studies confirm significant bias connected to self-reported gout. In our previous study conducted in 2004 [18], in which we also used the NHIRD as the primary data source, we found a much lower prevalence of gout (4.62%) than the estimate in 2005 in this study (5.91%). We consider that this disparity is primarily a result of the different lengths of observation (5 years in the previous study and 10 years in the present study) and therefore different degrees of identification of clinically silent patients. It is difficult to determine to what extent a shorter observation period might lead to underestimation of prevalence. In one case series, reported in 1961, the length of intercritical periods was less than 1 year in 62%, from 1 to 5 years in 27%, from 6 to 10 years in 4% and over 10 years in 7% of 614 patients [21]. Therefore, the gout prevalence in 2004 in our previous study could have been underestimated, because the length of the observation period was too short.

The incidence of gout has not been estimated previously in Taiwan, and incidence data in other countries also are relatively scarce. The largest population-based study was conducted in Rochester, MN, USA, In that study, researchers estimated annual incidences of 0.45 per 1,000 person-years and 0.62 per 1,000 person-years during the periods from 1977 to 1978 and 1995 to 1996, respectively [26]. In the Framingham study in the United States, researchers estimated an average annual incidence of 1.6 and 0.2 per 1,000 person-years for men and women, respectively, during the period between 1948 and 1980 [27], Those data are similar to the data reported for men in the John Hopkins Precursors Study, in which researchers found an incidence of 1.73 per 1,000 person-years in 1,216 male medical students over a median of 29 years [28]. Using data from the UK Second and Third National Studies of Morbidity in General Practice, the overall gout incidence was estimated to be 1.4 per 1,000 person-years in 1981 [29]. More recently, Mikuls *et al*. used the General Practice Research Database (GPRD) and estimated the UK all-age incidence of gout to be 1.31 cases per 1,000 person-years in 1999 [30]. Our recent estimate of UK gout incidence in 2012, using the same database, was 1.77 per 1,000 person-years [8]. However, all these estimates relate to Caucasians, and gout incidence has rarely been estimated in people of other ethnicities. Hochberg *et al*. reported that the incidence of gout in the period between 1958 and 1965 in African Americans was 3.11 per 1,000 person-years, which was 1.7-fold higher than that of Caucasians [28]. Similarly, the ARIC study researchers reported that the gout incidences in the period between 1987 and 2012 were 1.55 and 0.94 per 1,000 person-years for African American men and Caucasian American men, respectively [31]. To the best of our knowledge, our study is the first in which gout incidence has been reported in an Asian population. The incidence of gout in Taiwan was much higher than in other countries, suggesting significant racial and geographic variation in the aetiology of gout.

The age-specific incidence of gout is rarely reported. Data from the UK Second and Third National Studies of Morbidity in General Practice and the Framingham study, each undertaken in the 1980s, revealed that gout incidence peaked around the 50s age band [27,29]. More recently, Mikuls *et al*., who used the GPRD, found that gout incidence peaked later, between the ages of 65 and 84 years [30]. In our estimates in 2012, for which we used the same UK database, we also found a similar, later age of peak incidence [8]. In Taiwan, we have found that gout incidence peaks later, between the ages of 70 and 85 years. However, there was a significant difference in age-specific incidence between men and women. In men, we found a nonlinear distribution of age-specific incidence of gout, wherein a marked increase in gout incidence occurred in the two age bands of 20 to 39 and 50 to 69 years of age. In contrast, in women, age-specific incidence demonstrated a near-linear increase in the incidence of gout with age up to the peak incidence between ages 80 and 84 years. This appears unique compared with previous studies on age-specific incidence [27,29,30] in which investigators report a linear increase in the incidence of gout with age up to the peak and then a levelling off or slight decrease in both men and women thereafter. Our findings echo those of a previous study by Yu *et al*. which indicated that the onset of gout occurs earlier in Taiwanese [32] than in Caucasians [33] and Japanese [34]. The very high prevalence and incidence in Taiwan compared to that in China [35] and Japan [36], and the unique age-specific distribution of incidence, may partly reflect the composition of Taiwanese residents, a mixture of Han Chinese and indigenous people, who are known to be very prone to gout [11,37] and who are genetically related to Polynesians and Oceania/Pacific Islander populations [19]. This population stratification was also demonstrated by the regional variation in gout prevalence and incidence because the areas with the highest prevalence and incidence were those with the highest density of indigenous people with the highest genetic risk. However, genetic factors account for just one-third of phenotypic variation of gout in men and only one-fifth in women [18], so environmental factors could also contribute to the variable geographical distribution of gout in Taiwan. Further studies are required to address this issue.

In our present study, we found a trend of decreasing incidence, but generally a flattening of prevalence, during the study period. Several explanations may account for this finding. Firstly, relationships between incidence and prevalence are complex and involve other factors, such as disease duration and mortality [38]. In addition, the static relationship between prevalence and incidence is primarily based on a stable population with constant parameters [39]. Our study population was not stationary, and both prevalence and incidence were changing. Therefore, the change in incidence probably needs several years to translate to changes in prevalence. Secondly, the study period was probably too short, and longer observation would be required to measure long-term trends of prevalence and incidence accurately. Thirdly, NHI started in March 1995 in Taiwan, so the observation period in 2005 was 3 months short of 10 years of observation in estimates of other calendar years. This could have led to overestimation of incidence in 2005, the year showing the highest incidence of gout in the study period. The results of our study demonstrate that both prevalence and incidence were stationary after 2007, a period that is less liable to bias due to differential observation time. These results collectively show that gout prevalence and incidence were not increasing in the years studied, in contrast to the global trends. Further study is required to confirm our findings.

Regardless of the high prevalence of gout in Taiwan, the management of the disease remains poor. We found that only around one-third of prevalent gout cases involved patients who had contact with the health service in relation to their gout, and only one-fourth were actually prescribed ULT. We noticed no significant change in ULT prescription patterns during the study period, despite the publication of Taiwanese guidelines for the management of gout and hyperuricaemia in 2007 [40]. The poor management of gout seems to be a common problem globally [3,8,30,41-47]. Taking the United Kingdom as an example, the overall use of ULT in primary care has not changed in the past two decades, with only one-fourth to one-third of people with gout being given ULT [8,30]. Other deviations from recommended standards of care have also been reported in the United Kingdom [41,42,48]. These lines of evidence underscore the lack of knowledge and interest in gout among primary care physicians, which in turn reflects a number of varied barriers to optimal care of patients with gout [3,49-51]. However, in a recent proof-of-principle study conducted in the United Kingdom, researchers found that all patients with gout were willing to take long-term ULT once they were given full information on gout and its treatment [52]. In addition, these well-informed patients exhibited excellent adherence, and nine of ten patients achieved the therapeutic target at 1 year. Therefore, the essential part of optimising care of patients with gout is likely to be physician education to improve knowledge and promote interest in gout.

There are several limitations of the present study. Firstly, we based our case definition on physician-recorded diagnosis rather than according to American College of Rheumatology criteria [53], Rome [54] classification criteria or urate crystal identification. Secondly, we report 10-year period prevalence rather than lifetime prevalence, which theoretically would be higher. This is because of the inability to identify clinically silent patients who had no outpatient record of gout over the 10-year period. It is difficult to determine how many patients with gout were not included, because data on the length of asymptomatic intercritical gout periods are sparse. On the basis of estimates of the length of these intercritical gout periods in the case series reported by Yu *et al*. [21], an underestimation of 7% is probable. In addition to underestimation of prevalence, incidence would also be overestimated, as some incident gout cases could have included gout attacks prior to the 10-year observation period. We reported geographic variations of gout prevalence and incidence and attribute the significantly higher prevalence and incidence in the eastern coastal areas and offshore islets to aggregation of aboriginals; however, we do not have ethnicity data to support this notion. Certainly, variation in distribution of gout risk factors may also result in regional variation of gout prevalence and incidence. This hypothesis requires further study to be confirmed.

## Conclusions

In this population-based study, we found a high prevalence and incidence of gout in Taiwan, and the regional differences of gout prevalence and incidence were large. Uricosuric agents are more commonly prescribed than xanthine oxidase inhibitors. During the study period, the prevalence of gout was stable, whereas the incidence decreased, in Taiwan. Despite the high prevalence and incidence of gout in Taiwan, the management of gout remains poor, with only one-fourth of patients receiving ULT, which can potentially cure gout.

## Additional files

Additional file 1: Figure S1. Population pyramid of the general population in Taiwan in 2005. This figure describe the population structure in the general population in Taiwan in 2005. Additional file 2: Figure S2. Population pyramid of the general population in Taiwan in 2010. This figure describe the population structure in the general population in Taiwan in 2010.

## Abbreviations

APC: Annual percentage change
ARIC: Atherosclerosis Risk in Communities
CI: Confidence interval
GPRD: General Practice Research Database
NHI: National Health Insurance
NHIRD: National Health Insurance Research Database
ULT: Urate-lowering treatment
