# Supplementary figures and images for: Epidemiology and management of gout in Taiwan: a nationwide population study
Source: Arthritis Res Ther. 2015 Jan 23;17(1):13. doi: 10.1186/s13075-015-0522-8 (PMC4342824; doi:10.1186/s13075-015-0522-8)

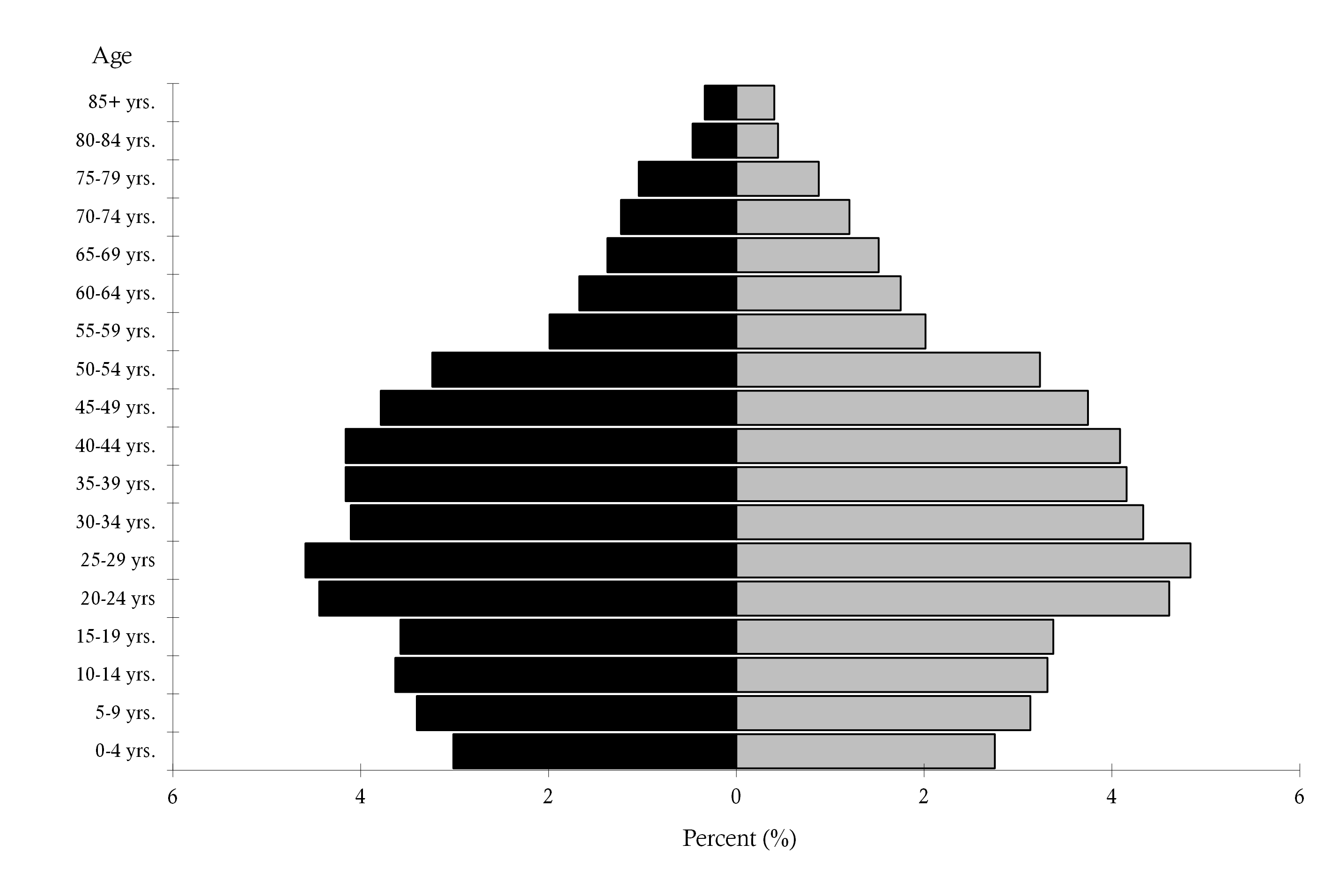

Supplement: Additional file 1: Figure S1. — Population pyramid of the general population in Taiwan in 2005. This figure describe the population structure in the general population in Taiwan in 2005. [file 13075_2015_522_MOESM1_ESM.tiff]

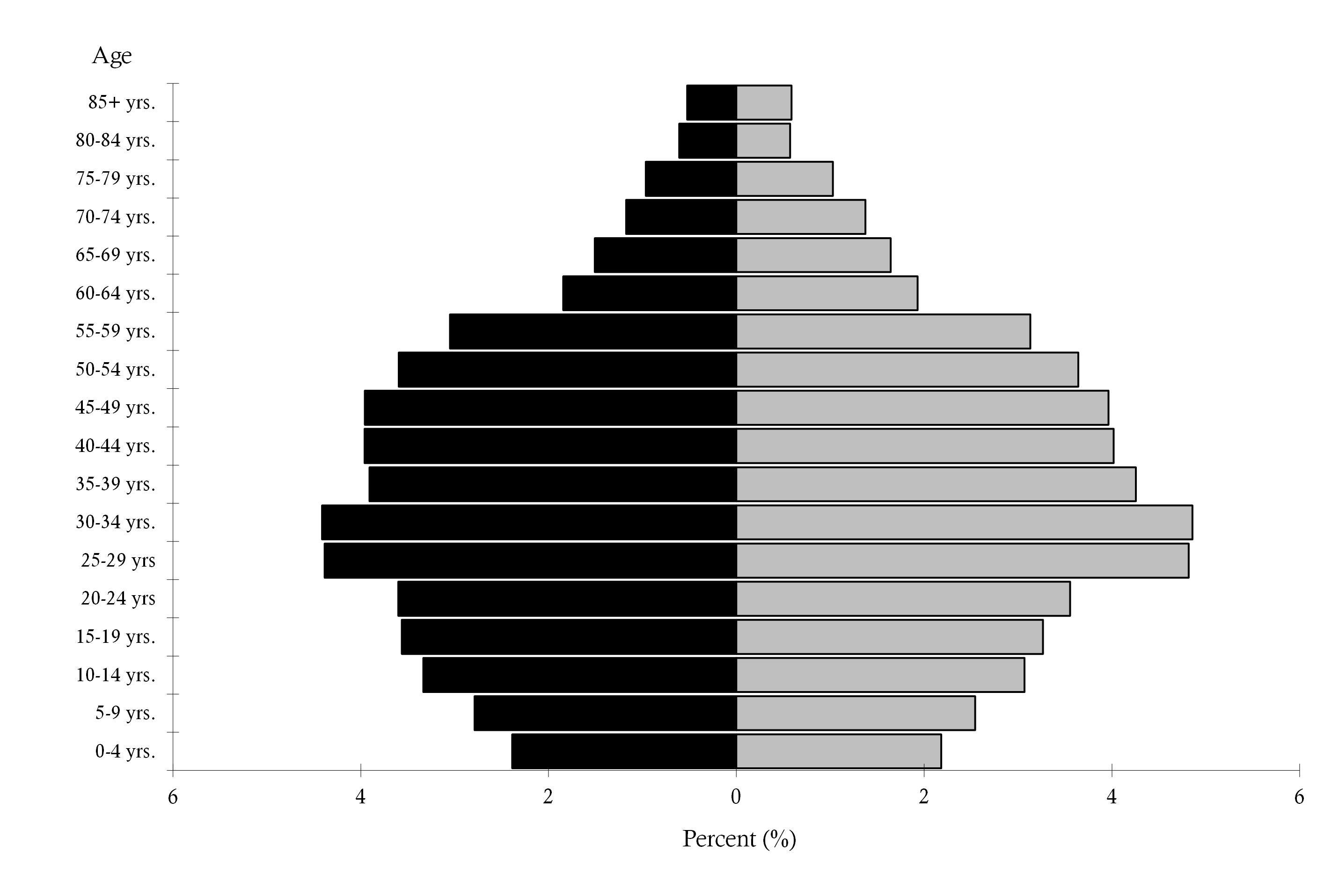

Supplement: Additional file 2: Figure S2. — Population pyramid of the general population in Taiwan in 2010. This figure describe the population structure in the general population in Taiwan in 2010. [file 13075_2015_522_MOESM2_ESM.tiff]
